# Computational Insights Into the Effects of the R190K and N121Q Mutations on the SARS-CoV-2 Spike Complex With Biliverdin

**DOI:** 10.3389/fmolb.2021.791885

**Published:** 2021-12-13

**Authors:** Zhiyuan Qu, Kaihang Li, Xiaoju Geng, Bo Huang, Jian Gao

**Affiliations:** ^1^ Jiangsu Key Laboratory of New Drug Research and Clinical Pharmacy, Xuzhou Medical University, Xuzhou, China; ^2^ Xuzhou Medical University Technology Transfer Center Co., Ltd., Xuzhou Medical University, Xuzhou, China

**Keywords:** SARS-CoV-2 spike, biliverdin, molecular dynamics simulation, MM/GBSA calculation, mutation

## Abstract

The SARS-CoV-2 spike has been regarded as the main target of antibody design against COVID-19. Two single-site mutations, R190K and N121Q, were deemed to weaken the binding affinity of biliverdin although the underlying molecular mechanism is still unknown. Meanwhile, the effect of the two mutations on the conformational changes of “lip” and “gate” loops was also elusive. Thus, molecular dynamics simulation and molecular mechanics/generalized Born surface area (MM/GBSA) free energy calculation were conducted on the wild-type and two other SARS-CoV-2 spike mutants. Our simulations indicated that the R190K mutation causes Lys190 to form six hydrogen bonds, guided by Asn99 and Ile101, which brings Lys190 closer to Arg102 and Asn121, thereby weakening the interaction energy between biliverdin and Ile101 as well as Lys190. For the N121Q mutation, Gln121 still maintained a hydrogen bond with biliverdin; nevertheless, the overall binding mode deviated significantly under the reversal of the side chain of Phe175. Moreover, the two mutants would stabilize the lip loop, which would restrain the meaningful upward movement of the lip. In addition, N121Q significantly promoted the gate loop deviating to the biliverdin binding site and compressed the site. This work would be useful in understanding the dynamics binding biliverdin to the SARS-CoV-2 spike.

## Introduction

Since the advent of novel severe acute respiratory syndrome coronavirus 2 (SARS-CoV-2), significant threats have been posed to the human population worldwide (Wang et al., 2020). Millions of infections and deaths have been caused by this severe epidemic (Hu et al., 2021). The spike protein trimers, a protruded structure that exists on the SARS-CoV-2 virions, are able to bind to a surface receptor on the cell and accommodate fusion of the viral and cellular membranes when they are glycosylated. For these reasons, with perfect conformational flexibility (Ke et al., 2020), the SARS-CoV-2 spike is a crucial viral antigen and the target in designing antibodies, which lead to its support for the current critical SARS-CoV-2 vaccine development efforts (Tregoning et al., 2020).

**FIGURE 1:**
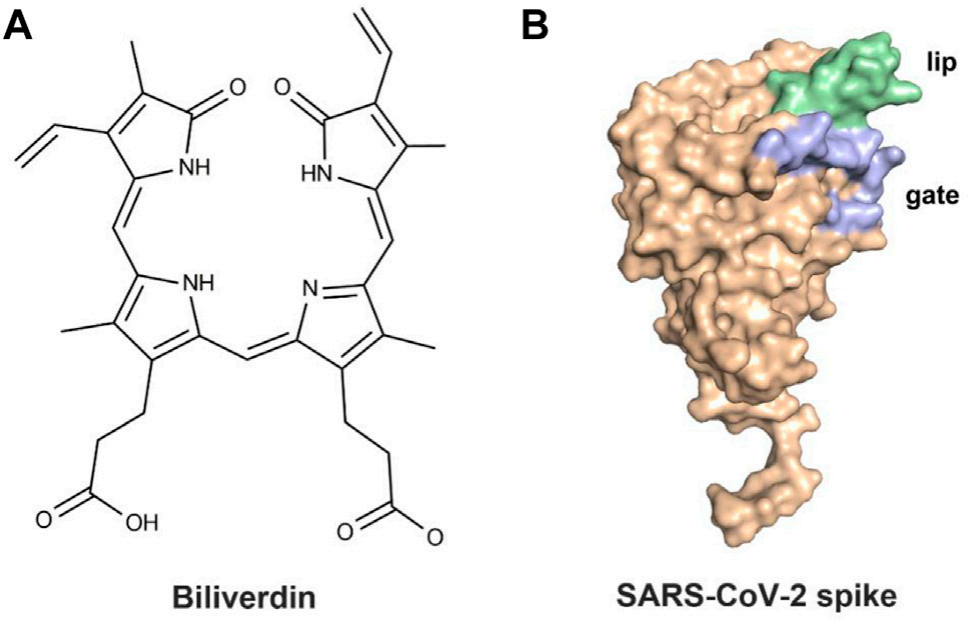
**(** **A** **)** Molecular structure of biliverdin and **(** **B** **)** the crystal structure of the SARS-CoV-2 spike.

The previous study (Rosa et al., 2021) revealed that the SARS-CoV-2 spike could bind biliverdin (Figure 1A), the tetrapyrrole product of heme metabolism. The tetrapyrrole interaction pocket to the spike N-terminal domain (NTD)’s deep cleft was mapped with the aid of cryo-electron microscopy and X-ray crystallography (Chi et al., 2020; Liu et al., 2020; Zost et al., 2020). The relevance between mutations in the SARS-CoV-2 spike NTD and viral escape from antibody immunity (Graham et al., 2021; Kemp et al., 2021) has been proven *via* observations in circulating viral strains (Tegally et al., 2021). The reactivity of SARS-CoV-2 spike with immune sera was intensively decreased by biliverdin. Meanwhile, a subset of neutralizing antibodies was also inhibited by biliverdin. Characterized neutralizing antibodies primarily bind the spike C-terminal domain (referred to as the receptor-binding domain, RBD) (Ju et al., 2020; Walls et al., 2020; Wrapp et al., 2020). The access to the dominant epitope of SARS-CoV-2 spike NTD can be controlled by an allosteric mechanism regulated through the recruitment of a metabolite.

After binding with biliverdin, a solvent-exposed loop “gate” in the wild type (WT) swings out of the way, allowing for Fab binding, which is complemented by an upward movement of a β-hairpin “lip” (Figure 1B) (Rosa et al., 2021). Moreover, the binding affinity of biliverdin bound to SARS-CoV-2 S1 is profoundly affected by the R190K and N121Q amino acid substitutions, with the corresponding K_d_ values significantly weakening (Rosa et al., 2021). To our knowledge, the dynamic effects of the two mutations on the lip and the gate loops have not been illustrated yet. Furthermore, the sharp decreased molecular mechanism of biliverdin binding affinity, caused by the two mutations, is still unknown. Accordingly, a combined strategy of molecular dynamics (MD) simulation and molecular mechanics/generalized Born (GB) surface area (MM/GBSA) free energy calculation was conducted on the complexes of wild-type and two mutated SARS-CoV-2 S1 proteins in the present study.

## Materials and Methods

### Construction of Simulation Systems

To figure out the impact of single-site mutations on the interactions of SARS-CoV-2 spike binding with biliverdin, three systems were constructed, including Spike^WT^/Biliverdin, Spike^N121Q^/Biliverdin, and Spike^R190K^/Biliverdin. In detail, the Spike^WT^/Biliverdin system was acquired from its corresponding X-ray crystal structure (PDB ID: 7b62), while the other two single-site mutations were constructed based on the system of Spike^WT^/Biliverdin in Sybyl-X2.1.

### MD Simulation and MM/GBSA Free Energy Calculation

With the aid of AMBER 12 (Case et al., 2005; Han et al., 2021) software, molecule energy minimization and MD simulation of the three systems were performed to reach the most stable conformation and decrease atomic energy. Some missing hydrogen atoms of the protein complex and ligand were added with the assistance of the tleap module in AMBER 12 software. The ligand biliverdin was minimized using the HF/6–31* optimization in the Gaussian 09 program (Frisch et al., 2016). In AMBER 12 software, the electrostatic potentials, derived from the restrained electrostatic potential (RESP) fitting technique in the Gaussian program, created the partial charges. Generated by the antechamber module in AMBER 12 (Wang et al., 2006), the field parameters and the partial charges for ligand biliverdin were established. The parameter for biliverdin was set by employing the general AMBER force field (GAFF) (Wang et al., 2004), and the standard AMBER force field (ff03) (Duan et al., 2003) was applied to define protein parameters in the following MD simulation.

Forming a rectangular box of TIP3P, the water molecules which extended 12 Å away from any solute atoms wrapped up the three systems. Appropriate numbers of K^+^ were added to neutralize those systems. Every single system was initially energy minimized *via* three steps with the help of a sander module, as described in a previous study (Chen H. et al., 2019). Then each system was heated gradually in the NVT ensemble from 0 to 300 K in 100 ps. Under a constant temperature of 300 K, a 200 ns MD simulation with a 2.0 fs time step was carried out for each system. Also, the SHAKE procedure was used to restrict all bonds involving at least one hydrogen atom. The particle mesh Ewald (PME) was used to deal with long-range electrostatic interactions. Concerning the following binding free energy calculation, the coordinates were saved every 100 ps in the sampling process.

With the aim of calculating the binding free energy, the calculation of MM/GBSA was carried out by using MM/GBSA in AMBER 12 software *via* the following equation (Kollman et al., 2000; Chen Q. et al., 2019; An et al., 2020; Shi et al., 2021)*.*

$$\begin{array}{l} \Delta G_{\mathrm{bind}}=G_{\mathrm{complex}}-G_{\mathrm{protein}}-G_{\mathrm{ligand}}\\ =\Delta E_{\mathrm{MM}}+\Delta G_{\mathrm{GB}}+\Delta G_{\mathrm{SA}}-T\Delta S\\ =\Delta E_{\mathrm{vdw}}+\Delta E_{\mathrm{ele}}+\Delta G_{\mathrm{GB}}+\Delta G_{\mathrm{SA}}-T\Delta S \end{array}$$

Δ*E*_MM_, the gas-phase interaction energy, is composed of two components: Δ*E*_vdw_ (van der Waals energy) and ΔE_ele_ (electrostatic energy) (Genheden and Ryde, 2015). Both Δ*G*_GB_ and Δ*G*_SA_ mean the components of the desolvation free energy. While the former is polar, the latter one is nonpolar. The polar desolvation free energy, with dielectric constants of the solvent and the solute set to 80 and 1, respectively, was calculated by the GB models developed by Onufriev et al. (2004). Normal-mode analysis was applied in evaluating the entropy contribution to the binding free energy.

## Results and Discussion

### Overall Structure and Dynamics

**FIGURE 2:**
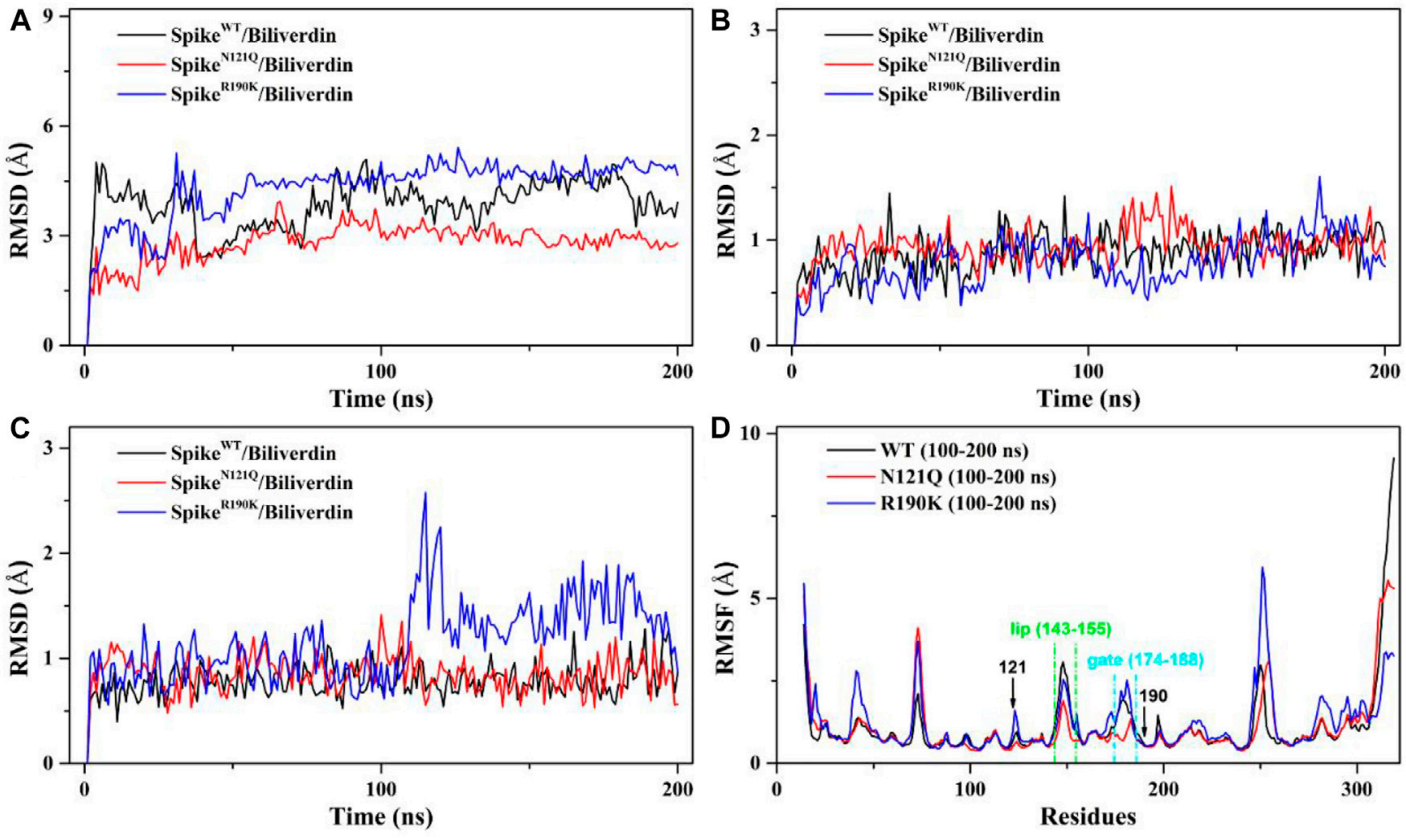
**(** **A** **)** RMSD of the backbone atoms in the three systems relative to their single initial structures. **(** **B** **)** RMSD of the backbone atoms in the “lip” region. **(** **C** **)** RMSD of the backbone atoms in the “gate” region. **(** **D** **)** RMSF of the backbone atoms in the three systems relative to their respective initial structures in 100–200 ns.

The root mean square deviation (RMSD) values of the whole protein backbone atoms were calculated to explore the conformation stability of three systems during the 200 ns MD simulation (Figure 2). The plot shows that all three systems reached equilibrium after 100 ns. The RMSD values of the Spike^WT^/Biliverdin and Spike^N121Q^/Biliverdin are around 3 Å, while that of the Spike^R190K^/Biliverdin mutation is around 4 Å (Figure 2A). Two single-site mutations do not induce significant conformation change to the protein since the RMSD values of two mutated systems stabilize in a very short time. The previous study [5] revealed that the SARS-CoV-2 spike contains the motion of loop regions including “gate” (residues 174–188) and “lip” (residues 143–155). To further assess the dynamic changes of two regions, the same RMSD calculation was conducted. Compared with the lip loop (Figure 2B), residues in the gate take on intensively small volatility and maintain a lower RMSD value, mostly below 1.0 Å (Figure 2C). It is noticeable that Spike^R190K^/Biliverdin fluctuates in both regions while Spike^WT^/Biliverdin and Spike^N121Q^/Biliverdin are quite stable. Three plots together proved that 200 ns was enough for three systems to relax.

We also employed the root mean fluctuation (RMSF) calculation of every amino acid residue based on a 100–200 ns MD trajectory to study the fluctuation of individual residues on the SARS-CoV-2 spike (Figure 2D). It can be clearly seen that the residues of Spike^N121Q^/Biliverdin and Spike^R190K^/Biliverdin have changed after comparative analysis. Especially at loop regions of the gate and lip, the amino acid residues of Spike^N121Q^/Biliverdin fluctuated less obviously than did those of Spike^WT^/Biliverdin and Spike^R190K^/Biliverdin in two regions. In line with the above RMSD analyses, Spike^R190K^/Biliverdin is the most unstable system as its RMSF value is much higher than the other two systems in most cases. However, for the lip loop as well as residues at positions 121 and 190, there is only a little difference in the fluctuation of the RMSF value in all three systems. Also, since the possible interaction with surrounding residues, the RMSF value of the gate loop in the Spike^N121Q^/Biliverdin is lower than those of the other two systems. In total, it is reasonable to carry out the following binding free energy calculation and free energy decomposition analysis based on the last 100 ns trajectories.

### Binding Free Energy Calculated by MM/GBSA

To analyze how the binding affinity between biliverdin and the SARS-CoV-2 spike is affected by two single-site mutations, MM/GBSA free energy calculation was conducted. According to Table 1, the binding free energy (Δ*G*_bind_) of Spike^WT^/Biliverdin (−18.60 kcal/mol) is stronger than that of Spike^R190K^/Biliverdin (−15.39 kcal/mol), which is in line with the fact that the R190K mutant would increase the Km value from 9.8 to 1,500 nM (Rosa et al., 2021). Unexpectedly, the calculated Δ*G*_bind_ of Spike^N121Q^/Biliverdin is −18.45 kcal/mol, comparable to the case of WT, which is different from the previous findings that the N121Q mutant is more sensitive to the binding interaction of biliverdin to SARS-CoV-2 spike (Rosa et al., 2021). With regard to the puzzling question, it will be discussed in the following binding mode analysis. Nevertheless, as for all three systems, the van der Waals interaction (Δ*E*_vdw_) plays a crucial role in the total binding free energy. Since the electrostatic interaction (Δ*E*_ele_) is completely counteracted by the polar desolvation energy (Δ*G*_GB_), the net of electrostatic interactions (Δ*E*_ele_ + Δ*G*_GB_) is even unfavorable to the binding affinities. Additionally, the differences in entropy contribution (TΔ*S*) of the three systems are not obvious.

**TABLE 1:** Binding free energies and individual energy terms of biliverdin in three systems calculated in MM/GBSA (kcal/mol).

System	Spike^WT^/Biliverdin	Spike^N121Q^/Biliverdin	Spike^R190K^/Biliverdin
Δ*E*_vdw_	−49.74 ± 2.26	−51.13 ± 2.69	−47.41 ± 2.71
Δ*E*_ele_	−93.82 ± 13.25	−116.72 ± 13.73	−79.64 ± 13.34
Δ*G*_GB_	117.03 ± 11.08	139.95 ± 11.81	103.19 ± 11.71
Δ*G*_SA_	−6.43 ± 0.45	−6.27 ± 0.68	−6.31 ± 0.54
TΔ*S*	−14.35 ± 3.14	−15.72 ± 3.25	−14.78 ± 3.16
Δ*G*_bind_	−18.60 ± 3.52	−18.45 ± 3.25	−15.39 ± 3.76
K_d_ (nM) (Rosa et al., 2021)	9.8	16,800	1,500

**FIGURE 3:**
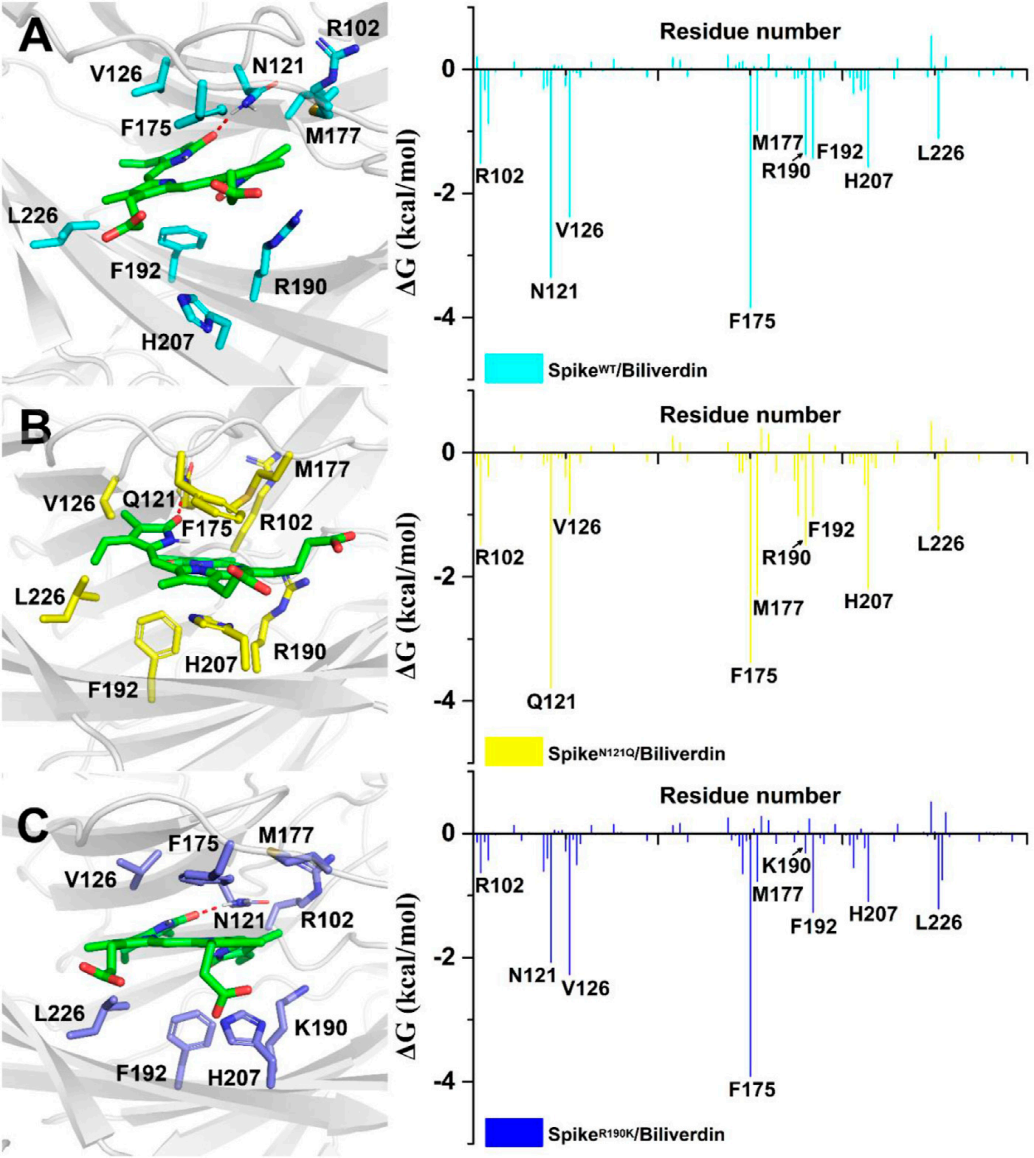
Residues around biliverdin and MM/GBSA decomposition results of the total binding free energies per residue for **(** **A** **)** Spike^WT^/Biliverdin, **(** **B** **)** Spike^N121Q^/Biliverdin, and **(** **C** **)** Spike^R190K^/Biliverdin.

To further investigate detailed binding modes between biliverdin and the SARS-CoV-2 spike, MM/GBSA free energy decomposition analysis that decomposes the total binding free energies into ligand–residue pairs is carried out accordingly. Eight residues, Asn121, Val126, Phe175, Met177, Arg190, Phe192, His207, and Leu226, play a dominant role in biliverdin binding, among which the side chain of Asn121 forms a hydrogen bond with biliverdin (Figure 3A). However, although the amino acid in position 121 mutated from asparagine to glutamine in Spike^N121Q^/Biliverdin, the side chain of Gln121 can still form a hydrogen bond with biliverdin although its contribution to the binding free energy changes slightly (Figure 3B). The binding pose of biliverdin in the Spike^N121Q^/Biliverdin changes greatly compared with the one in the Spike^WT^/Biliverdin. To be specific, biliverdin tends to approach His207 and away from Val126 since the length of the side chain at position 121 would become longer when the asparagine mutates to glutamine. In order to form the hydrogen bond, the biliverdin extends outside and deflects accordingly, whose dynamic procedure will be introduced in the following hydrogen bond analysis on the “gate” loop. For the Spike^R190K^/Biliverdin (Figure 3C), its binding mode is similar to the Spike^WT^/Biliverdin as the same amino acids contribute to the binding free energy. However, the contributions of Lys190 and Arg102 drop from −1.37 to −0.31 kcal/mol and from −1.58 to −0.70 kcal/mol, respectively.

### Uncover the Possible Molecular Mechanisms of Two Single-Site Mutations on the Binding Interaction Between the SARS-CoV-2 Spike and Biliverdin

**FIGURE 4:**
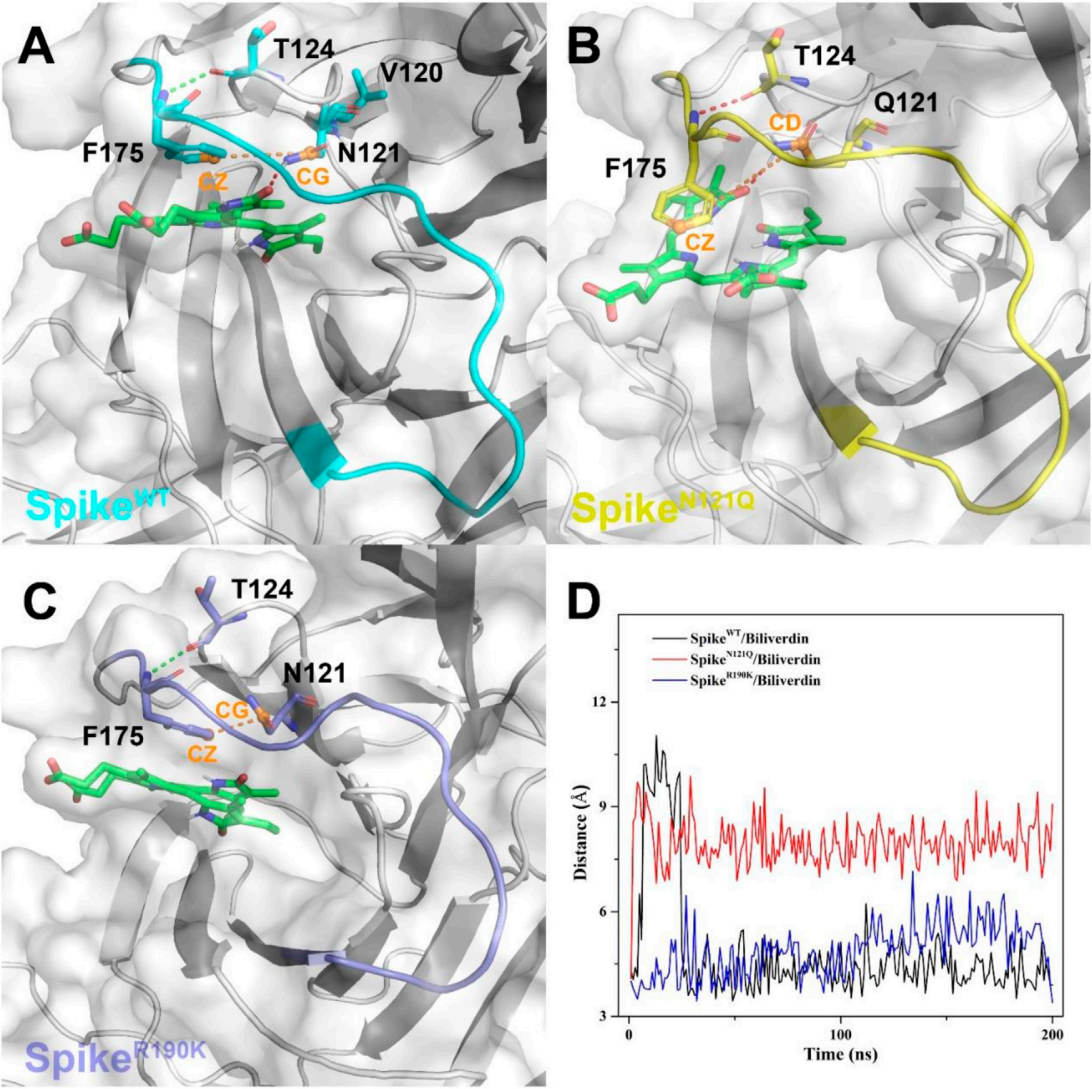
The binding interaction between the SARS-CoV-2 spike and biliverdin in **(** **A** **)** Spike^WT^/Biliverdin, **(** **B** **)** in Spike^N121Q^/Biliverdin, and **(** **C** **)** in Spike^R190K^/Biliverdin. **(** **D** **)** The distance between the CZ atom of Phe175 and the CG atom of Asn121 (or CD atom for the case of Gln121) was measured to reveal the deflection process of Phe175.

To further investigate how the N121Q mutant affects biliverdin binding affinity, we draw attention to hydrogen bonds around residue 121 in all three systems. In Spike^WT^/Biliverdin, one hydrogen bond is formed between the residue Asn121 and biliverdin. The side chain benzene ring of Phe175 can have a relative harmony π-π stack interaction with biliverdin (Figure 4A). After residue Asn121 mutates into Gln121, however, as the residue Gln121 side-chain elongates while the hydrogen bond between Gln121 and the biliverdin remains, the steric hindrance is generated between the side chains of Gln121 and Phe175, which makes the Phe175 side chain benzene ring deflect outward (Figure 4B). With two carboxyl groups exposed to the solvent, the biliverdin tends to move outward as the entire gate loop moves downward, narrowing the biliverdin binding pocket. Similar to that in Spike^WT^/Biliverdin, the gate loop does not change since the side chain benzene ring of residue Phe175 does not deflect in Spike^R190K^/Biliverdin (Figure 4C). To further verify the deflection process, we measure the distances between the CZ atom of Phe175 and the CG atom of Asn121 (or CD atom for the case of Gln121) (Figure 4D). It is not hard to find that for Spike^N121Q^/Biliverdin, the distance increased to 7.5 Å in a short time while the distances of the other two systems remained at 4.5 Å, which means that the deflection of Phe175 occurred very soon after the beginning of the dynamics simulation. The N121Q mutant likely leads the biliverdin binding as a differential pose when concerning the wild-type state. In other words, to counteract the unfavorable factor from the deflection of Phe175, compound biliverdin tends to be in an inactive state by inducted fit. In turn, the inducted-fit effect would result in the irreducible binding affinity of biliverdin to the SpikeN121Q protein.

**FIGURE 5:**
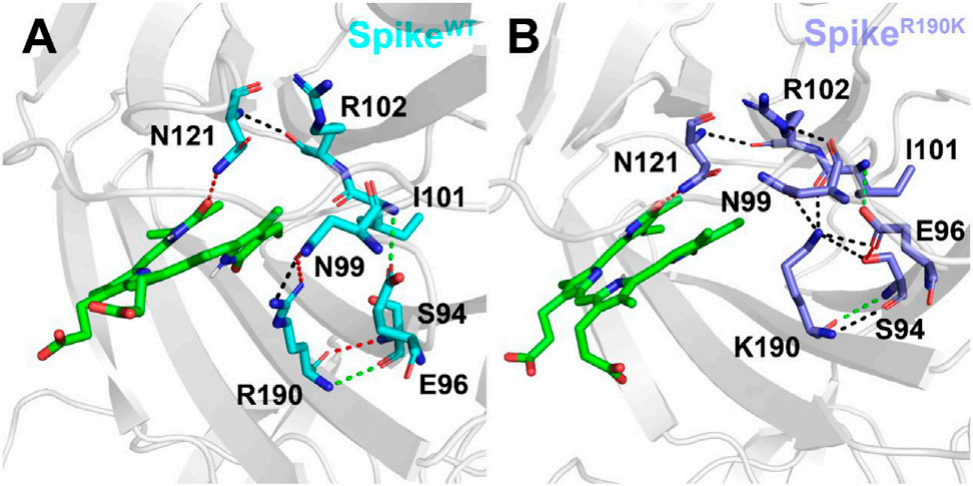
Hydrogen bond analysis around the amino acid at position 190 in the Spike^WT^/Biliverdin **(** **A** **)** and Spike^R190K^/Biliverdin **(** **B** **)**. Three different colors, red, green, and black, representing strong, moderate, and weak hydrogen bond interactions, were applied according to their probability range of >80%, 60%–80%, and 30%–60%, respectively.

As mentioned above, the mutation of R190K reduced the affinities of the biliverdin to Arg102 and Lys190. To explore the molecular mechanism, we also conducted a detailed analysis of hydrogen bonds near the amino acid at position 190, and the results are shown in Figure 5. It can be seen that in Spike^WT^/Biliverdin, Arg190 can form four hydrogen bonds with Asn99 and Ser94. Meanwhile, Asn121 forms one hydrogen bond with Arg102 in addition to one hydrogen bond with the compound biliverdin. According to Figure 5A, these two hydrogen bond networks are independent and unrelated, and the larger cavity enables the biliverdin to be deeply embedded and bound stably. In Spike^R190K^/Biliverdin (Figure 5B), however, Lys190 could be simultaneously integrated with Ser94, Glu96, Asn99, and Ile101, forming six hydrogen bonds in total. While the hydrogen bond between Asn121 and Arg102 continues to exist, Arg102 also forms hydrogen bonds with Asn99. Guided by Asn99 and Ile101, Lys190 is brought closer to Arg102 and Asn121, which leads to the narrow cavity formed by these amino acids and is not conducive to the compound biliverdin insertion, widening the distances between biliverdin and Ile101 as well as Lys190 and reducing their respective energy contributions.

### Effect of Two Single-Site Mutations on the Conformations of “Lip” and “Gate” by Hydrogen Bond Analysis

Previous research (Rosa et al., 2021) has already validated that the antibody binding to the SARS-CoV-2 spike NTD is inhibited by biliverdin *via* an allosteric mechanism which is associated with two loop regions of “lip” and “gate”. It will be meaningful to study the possible conformation of both the two loop regions in Spike^WT^/Biliverdin and the two mutants. Thus, hydrogen bond analyses were conducted on the two loops based on the last 100 ns trajectories (Supplementary Table S1). The cutoff length value of forming hydrogen bonds is set at 3 Å. For a clearer observation, three different colors, red, green, and black, representing strong, moderate, and weak hydrogen bond interactions, respectively, were applied according to their probability range of >80%, 60%–80%, and 30%–60%, respectively.

**FIGURE 6:**
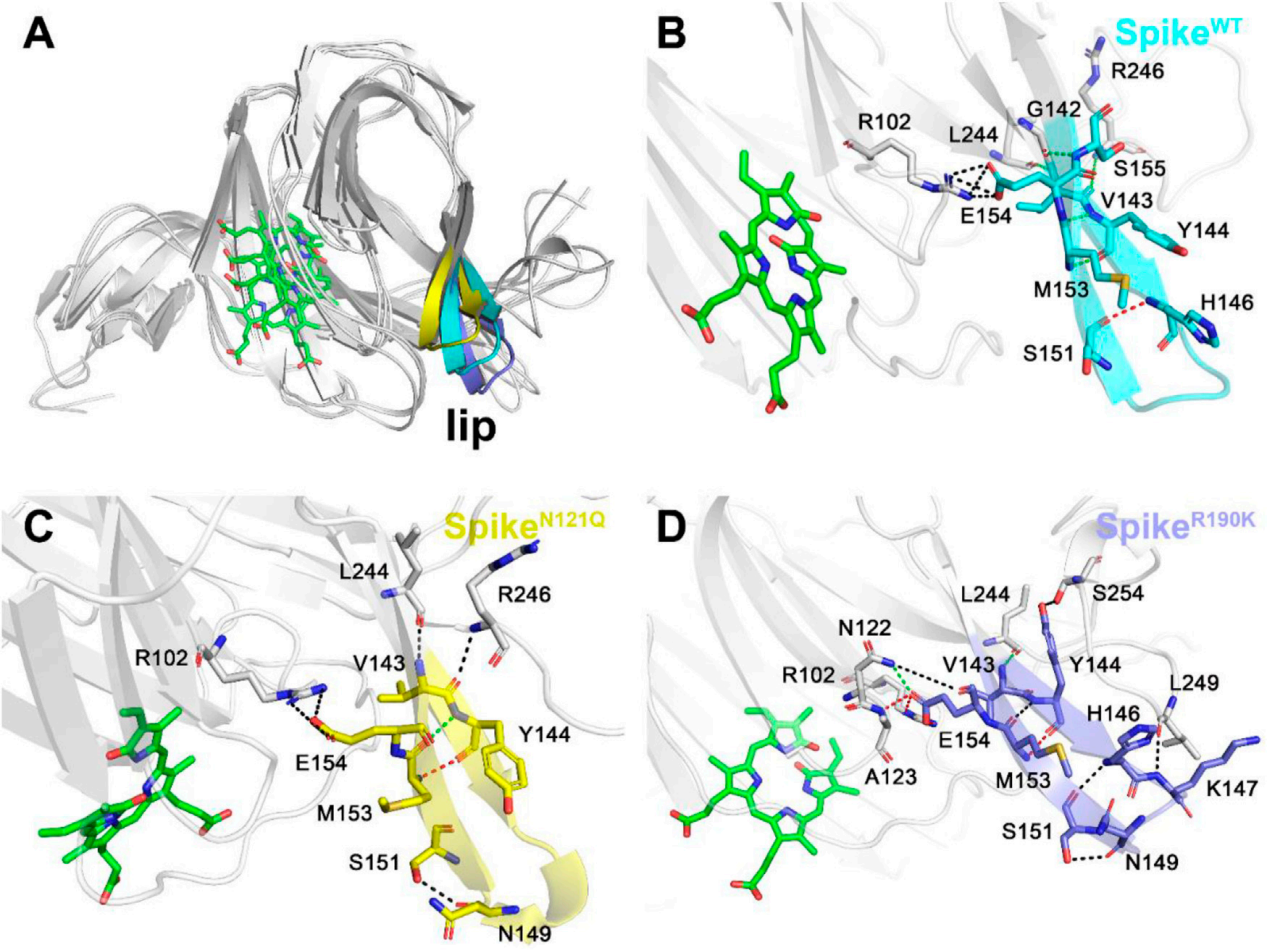
Three different colors, red, green, and black, representing strong, moderate, and weak hydrogen bond interactions, were applied according to their probability range of >80%, 60%–80%, and 30%–60%, respectively. **(** **A** **)** Comparison of the lip loops in three systems. **(** **B** **)** Illustration of the hydrogen bond interaction of the lip in Spike^WT^/Biliverdin, **(** **C** **)** in Spike^N121Q^/Biliverdin, and **(** **D** **)** in Spike^R190K^/Biliverdin.

After observing three systems overlapping together (Figure 6A), it can be concluded that two single-site mutations have posed significant changes to the lip region. As for the lip of Spike^WT^/Biliverdin (Figure 6B and Supplementary Figure S1A), one strong hydrogen bond is formed between His146 and Ser151, and five moderate hydrogen bonds are formed between Met153 and Tyr144 (two hydrogen bonds are formed), Gly142 and Ser155, Val143 and Arg246, and Leu244 and Val143 while Arg102 and Glu154 form four weak hydrogen bonds. Concerning Spike^N121Q^/Biliverdin (Figure 6C), the advent of residues Leu244 and Arg246 resulted in a new hydrogen bond formed each with Val143 and Tyr144, leading to the loop_244-261_ approaching the lip (Figure 6C and Supplementary Figure S1B). Moreover, compared with Spike^N121Q^/Biliverdin, new hydrogen bonds form between Asn122 and Glu154, Ala123 and Glu154, Ser254 and Tyr144, and Leu249 and Lys147 in the Spike^R190K^/Biliverdin (Figure 6D); it reveals that not only loop_244-261_ but also loop_122-123_ interacts with the lip (Supplementary Figure S1C). The stabilized lip is likely to exist in two mutated systems, which would restrain the meaningful upward movement of the lip as discussed in previous literature (Rosa et al., 2021), ultimately interfering with the antibody fixing on the SARS-CoV-2 spike.

**FIGURE 7:**
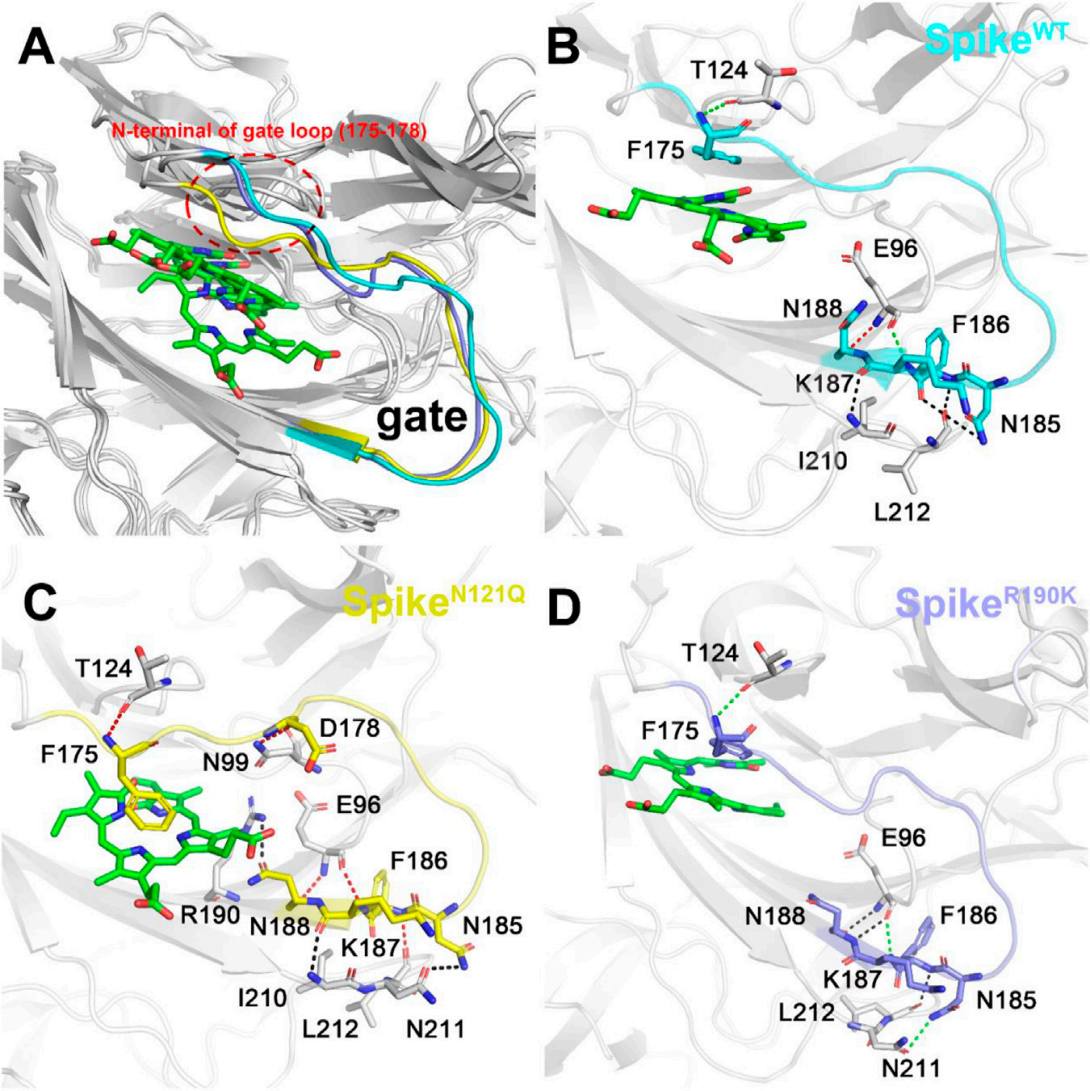
Three different colors, red, green, and black, representing strong, moderate, and weak hydrogen bond interactions, were applied according to their probability range of >80%, 60%–80%, and 30%–60%, respectively. **(** **A** **)** Comparison of the gate loops in three systems. Illustration of hydrogen bond interaction of the lip **(** **B** **)** in Spike^WT^/Biliverdin, **(** **C** **)** in Spike^N121Q^/Biliverdin, and **(** **D** **)** in Spike^R190K^/Biliverdin.

The possible conformation changes of the gate loop derived from two mutants were also studied (Figure 7). It is not hard to find that the N121Q mutation had the N-terminal residues of the gate (residues 175 to 178) dramatically changed in contrast to the other two systems (Figure 7A). The hydrogen bond network of the Spike^WT^/Biliverdin system is characterized by one strong hydrogen bond formed between Asn188 and Glu96, two moderate ones formed between Glu96 and Lys187 and between Phe175 and Thr124, and another three weak hydrogen bonds (Figure 7B). Intriguingly, a strong Asn99–Asp178 hydrogen bond is formed in Spike^N121Q^/Biliverdin (Figure 7C), which triggers the gate loop approaching and narrowing the binding pocket of the biliverdin. As for Spike^R190K^/Biliverdin (Figure 7D), an additional moderate hydrogen bond is formed by Asn185 and Asn211 to stabilize the similar gate conformation in Spike^WT^/Biliverdin.

## Conclusion

The global spread of the novel coronavirus disease 2019 (COVID-19), one of the deadliest pandemics in modern history, has been unprecedented since it first emerged. The SARS-CoV-2 spike is currently the main target of antibody design. Previous studies have reported the crystal structure of the SARS-CoV-2 spike and biliverdin and have come up with the idea that two single-site mutations, R190K and N121Q, will weaken the binding affinity of the biliverdin although the potential molecular mechanism is still unknown. As a result, this project studied the WT and two other mutants of the SARS-CoV-2 spike, employing MD simulation and MM/GBSA free energy calculation. Our simulations confirmed that the R190K mutation causes amino acid 190 to form six hydrogen bonds with surrounding residues Ser94, Glu96, Asn99, and Ile101, which, guided by Asn99 and Ile101, brings Lys190 closer to Arg102 and Asn121, thereby weakening the interaction energy between biliverdin and Ile101 as well as Lys190. However, in the case of N121Q mutation, although Q121 still maintained hydrogen bond interaction with biliverdin, the overall binding mode deviated significantly under the reversal of the benzene ring of the Phe175 side chain compared with WT. Moreover, we found that R190K and N121Q mutants would stabilize the lip loop. The stabilized lip was likely to exist in two mutated systems, which would restrain the meaningful upward movement of the lip, ultimately interfering with the antibody fixing on the SARS-CoV-2 spike. In addition, N121Q significantly promoted the gate loop deviating to the biliverdin binding site and compressed the site so that biliverdin could not maintain the binding mode of WT. However, the R190K mutation had little influence on the gate loop and could also make the structural change of the gate loop similar to WT. Studying the interaction between the SARS-CoV-2 spike and the biliverdin and two single-site mutations using simulation methods, we hope, will support future antibody research targeting the SARS-COV-2 spike.

## Data Availability

The original contributions presented in the study are included in the article/Supplementary Material, further inquiries can be directed to the corresponding author.
