# Supplementary material for: Computational Insights Into the Effects of the R190K and N121Q Mutations on the SARS-CoV-2 Spike Complex With Biliverdin
Source: Front Mol Biosci. 2021 Dec 13;8:791885. doi: 10.3389/fmolb.2021.791885 (PMC8711121; doi:10.3389/fmolb.2021.791885)
Supplement: Supplementary file 1 [file DataSheet1.docx]

Supplementary Material

**Computational insights into the effects of the R190K and N121Q mutations on the SARS-CoV-2 spike complex with biliverdin**

Zhiyuan Qu^a^, Kaihang Li^a^, Xiaoju Geng^a^, Bo Huang^a^ and Jian Gao^a,b,^*

*^a^Jiangsu Key Laboratory of New Drug Research and Clinical Pharmacy,* *Xuzhou Medical University, Xuzhou, Jiangsu 221004, P. R. China*

*^b^Xuzhou Medical University Technology Transfer Center Co., Ltd., Xuzhou Medical University, Xuzhou, Jiangsu 221004, P. R. China*

*****Correspondence: gaojian@xzhmu.edu.cn; Tel.: +86-0516-8326-2137

**
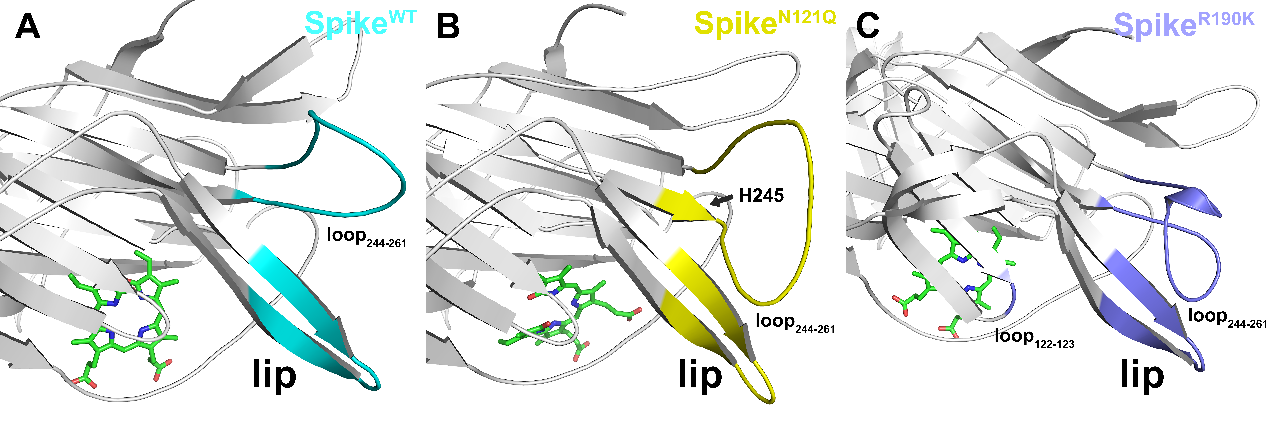
**

**Figure S1.** Comparison of the lip loops in Spike^WT^/Biliverdin (A), Spike^N121Q^/Biliverdin (B), and Spike^R190K^/Biliverdin (C).

**Table S1.** Hydrogen bond analyses of "lip" and "gate" loop regions in three systems calculated from last 100 ns trajectories.

|  | **Acceptor** | **DonorH** | **Donor** | **Frac (%)** | **Dist (Å)** |
| --- | --- | --- | --- | --- | --- |
| Spike^WT^/Biliverdin | S151@O | H146@H | H146@N | 90.0 | 2.84 |
|  | Y144@O | M153@H | M153@N | 70.0 | 2.86 |
|  | M153@O | Y144@H | Y144@N | 67.5 | 2.89 |
|  | G142@O | S155@H | S155@N | 65.0 | 2.86 |
|  | V143@O | R246@H | R246@N | 62.5 | 2.86 |
|  | L244@O | V143@H | V143@N | 62.5 | 2.90 |
|  | D178@O | N99@HD22 | N99@ND2 | 60.0 | 2.86 |
|  | E154@OE2 | R102@HH12 | R102@NH1 | 52.5 | 2.77 |
|  | L212@O | F186@H | F186@N | 50.0 | 2.84 |
|  | F186@O | N185@HD22 | N185@ND2 | 47.5 | 2.86 |
|  | K187@O | I210@H | I210@N | 47.5 | 2.89 |
|  | E154@OE2 | R102@HH22 | R102@NH2 | 42.5 | 2.80 |
|  | E154@OE1 | R102@HH22 | R102@NH2 | 32.5 | 2.77 |
| Spike^N121Q^/Biliverdin | E96@O | K187@H | K187@N | 87.5 | 2.84 |
|  | L212@O | F186@H | F186@N | 85.0 | 2.82 |
|  | T124@O | F175@H | F175@N | 82.5 | 2.82 |
|  | N188@O | E96@H | E96@N | 82.5 | 2.81 |
|  | Y144@O | M153@H | M153@N | 80.0 | 2.85 |
|  | D178@O | N99@HD22 | N99@ND2 | 80.0 | 2.82 |
|  | M153@O | Y144@H | Y144@N | 77.5 | 2.86 |
|  | N188@OD1 | R190@HH21 | R190@NH2 | 55.0 | 2.85 |
|  | V143@O | R246@H | R246@N | 55.0 | 2.88 |
|  | N211@OD1 | N185@HD22 | N185@ND2 | 52.5 | 2.84 |
|  | L244@O | V143@H | V143@N | 50.0 | 2.87 |
|  | E154@OE1 | R102@HH12 | R102@NH1 | 45.0 | 2.76 |
|  | K187@O | I210@H | I210@N | 37.5 | 2.87 |
|  | E154@OE2 | R102@HH22 | R102@NH2 | 35.0 | 2.84 |
|  | N149@O | S151@HG | S151@OG | 35.0 | 2.84 |
| Spike^R190K^/Biliverdin | E154@OE1 | A123@H | A123@N | 97.5 | 2.83 |
|  | E154@OE2 | R102@HH22 | R102@NH2 | 90.0 | 2.81 |
|  | E154@OE1 | R102@HH12 | R102@NH1 | 90.0 | 2.79 |
|  | Y144@O | M153@H | M153@N | 85.0 | 2.84 |
|  | T124@O | F175@H | F175@N | 77.5 | 2.82 |
|  | N211@OD1 | N185@HD21 | N185@ND2 | 70.0 | 2.83 |
|  | L244@O | V143@H | V143@N | 70.0 | 2.88 |
|  | E154@OE1 | N122@HD22 | N122@ND2 | 67.5 | 2.82 |
|  | E96@O | K187@H | K187@N | 65.0 | 2.86 |
|  | S254@OG | Y144@HH | Y144@OH | 57.5 | 2.83 |
|  | S151@O | H146@H | H146@N | 55.0 | 2.86 |
|  | M153@O | Y144@H | Y144@N | 55.0 | 2.85 |
|  | E96@O | N188@H | N188@N | 52.5 | 2.92 |
|  | N188@O | E96@H | E96@N | 52.5 | 2.86 |
|  | L212@O | F186@H | F186@N | 42.5 | 2.87 |
|  | E154@O | N122@HD21 | N122@ND2 | 40.0 | 2.87 |
|  | L249@O | K147@H | K147@N | 37.5 | 2.92 |
|  | N149@O | S151@HG | S151@OG | 30.0 | 2.73 |
